# Managing In- and Out-Migration of Health Workforce in Selected Countries in South East Asia Region

**DOI:** 10.15171/ijhpm.2017.49

**Published:** 2017-05-08

**Authors:** Viroj Tangcharoensathien, Phyllida Travis, Achmad Soebagjo Tancarino, Krisada Sawaengdee, Yanchen Chhoedon, Safeenaz Hassan, Nareerut Pudpong

**Affiliations:** ^1^International Health Policy Program, Ministry of Public Health, Nonthaburi, Thailand.; ^2^WHO South East Asia Region, Delhi, India.; ^3^Ministry of Health, Jakarta, Indonesia.; ^4^Public Health Technical Office, Ministry of Public Health, Nonthaburi, Thailand.; ^5^Ministry of Health, Thimphu, Bhutan.; ^6^Ministry of Health, Malé, Maldives.; ^7^Healthcare Accreditation Institute (Public Organization), Nonthaburi, Thailand.

**Keywords:** Management, In-Migration, Out-Migration, Health Workforce, International, Recruitment, Asia

## Abstract

**Background:** There is an increasing trend of international migration of health professionals from low- and middle- income countries to high-income countries as well as across middle-income countries. The WHO Global Code of Practice on the International Recruitment of Health Personnel was created to better address health workforce development and the ethical conduct of international recruitment. This study assessed policies and practices in 4 countries in South East Asia on managing the in- and out-migration of doctors and nurses to see whether the management has been in line with the WHO Global Code and has fostered health workforce development in the region; and draws lessons from these countries.

**Methods:** Following the second round of monitoring of the Global Code of Practice, a common protocol was developed for an in-depth analysis of (a) destination country policy instruments to ensure expatriate and local professional quality through licensing and equal practice, (b) source country collaboration to ensure the out-migrating professionals are equally treated by destination country systems. Documents on employment practice for local and expatriate health professionals were also reviewed and synthesized by the country authors, followed by a cross-country thematic analysis.

**Results:** Bhutan and the Maldives have limited local health workforce production capacities, while Indonesia and Thailand have sufficient capacities but are at risk of increased out-migration of nurses. All countries have mandatory licensing for local and foreign trained professionals. Legislation and employment rules and procedures are equally applied to domestic and expatriate professionals in all countries. Some countries apply mandatory renewal of professional licenses for local professionals that require continued professional development. Local language proficiency required by destination countries is the main barrier to foreign professionals gaining a license. The size of outmigration is unknown by these 4 countries, except in Indonesia where some formal agreements exist with other governments or private recruiters for which the size of outflows through these mechanisms can be captured.

**Conclusion:** Mandatory professional licensing, employment regulations and procedures are equally applied to domestic and foreign trained professionals, though local language requirements can be a barrier in gaining license. Source country policy to protect their out-migrating professionals by ensuring equal conditions of practice by destination countries is hampered by the fact that most out-migrating professionals leave voluntarily and are outside government to government agreements. This requires more international solidarity and collaboration between source and destination countries, for which the WHO Global Code is an essential and useful platform.

## Background

There is an increasing trend of international migration of health professionals from low- and middle-income countries to high-income countries.^1^ Health personnel outflow from countries in South East Asia (SEA) to countries in Economic Co-operation and Development (OECD) is significant.^1^ Indian born doctors rank top in terms of numbers of foreign health workers in OECD countries. Sri Lanka has the highest expatriate rate: one-third of the total doctors trained in Sri Lanka are practicing in the OECD.^2^ Even Timor Leste, which has very few doctors, is contributing to the health workforce in OECD countries: 35 Timor Leste doctors were reported to be practicing in OECD countries, equivalent to 30.7% of the total stock working in Timor, a significant loss.^2^ There is also international migration of health workers to countries in the Gulf Cooperation Council (GCC) but the picture is different, as source countries include both developed and developing nations.^5^

The motivation for health professionals from developing countries to move to developed (‘destination’) countries is mainly their need for higher salaries, better work conditions and career advancement. At the same time, push factors in their home (or ‘source’) countries are often not adequately addressed. The unintended consequence is unmet health needs – especially among the poor and rural populations - in source countries.^3^

A number of studies have examined health worker migration since the 2006 World Health Report Working Together for Health.^4-6^ Kingma provided a comprehensive picture of the complexity of international migration of nursing personnel in 2007, showing that OECD countries relied heavily on foreign trained nurses. For example 30 000 nurses and midwives educated in sub-Saharan Africa were employed in seven OECD countries. At the same time, paradoxically, sub-Saharan African countries had a critical shortage of health workers, and the governments did not have sufficient budgets to employ their domestically trained staff. This resulted in a large number of unemployed nurses seeking jobs abroad. Hammett^6^ explored physician migration across the global south, while Yeates and Pillinger^7^ noted that apart from the WHO Global Code of Practice on the International Recruitment of Health Personnel in 2010, knowledge about the nature and range of global policy actors, policy responses and initiatives in human resources for health (HRH) migration remained very limited. They argued for better monitoring of migration flows and of ethical recruitment practices.

The WHO Global Code of Practice on the International Recruitment of Health Personnel (hereafter called the Code), was created to better address health workforce development and the ethical conduct of international recruitment. It is a global framework for international cooperation and was adopted in May 2010.^8^ The Code was developed because the problems of and solutions to international health workforce migration are inter-connected and require collective action across source and destination countries. The Code aims to ensure that migrant health workers are treated fairly, and to ensure that source countries with health workforce shortages are not further depleted by the growing needs of rich countries. Since 2010 there have been 2 rounds of monitoring of progress on its implementation.

The number of countries that participated in the first progress report in 2013 was disappointing: only 56 out of 194 WHO Member States (29%) reported.^9^ Most of these were destination countries, with 71% being from the WHO European region. A key finding was that migrant health professionals in most countries had the same legal rights and employment conditions as domestically trained health workers. The main reason for low reporting was lack of awareness of the Code by WHO Member States. Only 3 out of 11 countries from WHO South-East Asia Region (Indonesia, Maldives and Thailand) reported.

The reviews of literature pose several policy questions. There is little in-depth analysis of how countries in the SEA Region are addressing international health worker out-migration, especially when they already face health worker shortages. Current policy questions include how are countries with different levels of health workforce density managing to fulfill their national health workforce needs – is it through producing more health workers, or encouraging in-migration and ensuring the professional standards of expatriate workers? Or both? How do source countries ensure their out-migrating professionals are equally treated in destination countries?

This paper reviews how policies in 4 countries in SEA are being applied in order to ensure; consistent professional standards and equal practice opportunities for expatriate professionals, their out-migrating professionals are equally treated in destination countries, and continued professional education and relicensing for domestic and expatriate professionals is provided.

## Methods

The second progress report on implementing the Code was carried out in 2015. This time, 6 countries from WHO’s SEA Region reported: Bangladesh, Bhutan, Indonesia, Maldives, Myanmar, and Thailand. WHO convened a meeting of the 6 countries to discuss progress and challenges with implementing the Code. A common protocol to do a more in-depth analysis of the policy instruments being used was developed and agreed to by the 6 countries. The protocol covered national health workforce context; policies and regulations concerning the rights, terms of employment, recruitment practices and conditions of work for domestically and foreign trained health workers working in-country; licensing and relicensing practices to ensure quality; and policies in place to protect out-migrating professionals, to ensure they are treated the same as domestically trained health workers in destination countries.

Four country reports prepared using this protocol provided sufficient information for a qualitative cross-country analysis of policy and practice: Bhutan, Maldives; Indonesia, and Thailand. For the 4 countries, their policies and practices as both a source and a destination country for health professionals are considered.

## Results

### Health Workforce Context in the 4 Countries

Bhutan and the Maldives both have populations of under 1 million.^10^ Neither country can provide domestic training for their doctors, but both have in-country training of nurses and midwives. Both Governments recruit secondary school students to study medicine, fully financed by the government, in other countries in the Region - on the condition that they return to serve in the country’s health service once they graduate. All Bhutanese medical graduates from schools outside the country currently return to serve the government health service. However, some Maldivian medical graduates do not return home, despite the existence of contracts with government.

Bhutan faces a critical shortage of health workers. Together, the ratio of doctors, nurses and midwives is 1.24 per 1000 population,^11^ below the global threshold of 2.28 per 1000 population.^12^ Based on the National Health Service Standard 2015, projections estimate that Bhutan requires 194 additional doctors, of which 83 are general practitioners and 111 specialists, and additional 658 nurses to fill the gap by 2020.

The Maldives does not face a health workforce shortage: there are 6.46 doctors, nurses and midwives per 1000 population, much above the 2.28 indicative threshold. However, the Maldives relies heavily on immigrant doctors: of the total 489 doctors in 2012, 85% were expatriates, while expatriates are 55% of a total of 1911 nurses.^13^ Maintaining a stable number of doctors to meet country health needs is the main challenge.

Indonesia and Thailand have adequate capacity for in-country training of doctors and nurses. Indonesia has successfully increased its production capacity, particularly of nurses and midwives. Annual production capacity is now over 6400 doctors and over 40 000 nurses. In 2015, doctors, nurse and midwives density was 3.22 per 1000 population, above the indicative threshold, therefore Indonesia does not face critical shortages but mal-distribution remained a critical issue.^14^ For example, in 2015, there were 11 specialists serving one million people in East Nusa Tenggara; while there were 148 specialists per million in Yogyakarta – a 13 fold variation, hampering equitable access to specialist care **(**Table 1).

**Table 1:** Five Categories of Medical Specialist Per Million Populations in 3 Selected Province, Indonesia, 2015

**Province**	**Population (million)**	**Pediatrician**	**Surgeon**	**Internist**	**OB-GYN**	**Anesthesiologist**	**Specialists (per million)**
East Nusa Tenggara	4.7	11	12	15	12	2	11
East Kalimantan	3.5	38	57	43	57	22	62
Yogyakarta	3.5	119	109	125	109	56	148

Indonesia does not rely on foreign doctors and nurses. By 2015, there were 103 745 doctors and 824 000 nurses registered with the Medical Council and National Nurses Association respectively. Of these, there were only 106 doctors and 8 nurses who are foreign professionals. In fact, a surplus of nurse production has led to a government policy to seek employment opportunities abroad, such as to countries in the Middle East and Asia.

Thailand has neither shortage nor surplus of health personnel, though there are some remaining problems of mal-distribution. The density of physicians and nurses was 2.47 per 1000 population, while the number of allied health personnel has also increased. There are 19 medical schools (of which only one is privately owned) and 80 nursing schools (21 being privately owned). In 2015, medical schools produced 2500 medical graduates; nursing schools supply about 9800 graduates.

All 4 countries have committed to progressing towards universal health coverage (UHC), though each at a different pace and through different approaches. Bhutan covers the whole population through public financing of free government health services. The Maldives provides free care to the whole population, with funds channeled through a national health insurance agency. Thailand has achieved full population coverage since 2002. Indonesia has achieved very high population coverage although there are supply side limitations in certain geographical areas. Table 2 provides a snapshot of the 4 selected countries in SEA.

**Table 2:** At a Glance, 4 Selected Countries in SEA, Circa 2010

	**Bhutan**	**Indonesia**	**Maldives**	**Thailand**
GNI per capita, US$	2390	3650	7290	5410
Population size, million	0.8	260	0.4	64
Births attended by skilled staff, % of total	80	83	99	100
Total medical schools	0	75	0	19
Public/private	0/0	33/42	0/0	18/1
Total nursing schools	2	909	1	80
Public/private	2	60/ 849	1/0	59/21
Total doctor in the pool	249	103 745	489	38 168
Foreign doctors	29	106	415	47^a^
Total nurse personnel	1085	824 000	1911	140 620
Foreign nurses	0	8	1047	10^a^
Annual production capacities				
Doctors	20^c^	7305	14^d^	2500
Nurses	75^b^	28 825	118	9800^e^
Midwives	NA	18 545	12	NA

Abbreviation: SEA, South East Asia ^a^ Native born-foreign trained. ^b^ General nurse and midwife. ^c^ Total number of Bhutanese medical doctors trained abroad and funded by the government. ^d^ Total number of Maldivian medical students trained abroad and funded by the government. ^e^ Total number of registered nurses who are trained in one curriculum of nursing and midwifery, Thailand does not train stand alone midwife.

Several policy instruments are being used, which are in line with recommendations of the Code: policies by destination countries to ensure expatriate professional quality through licensing and equal practice, and source country actions to ensure their out-migrating professionals are equally treated by destination country systems.

### Policies as Destination Countries on Licensing and Re-licensing of Expatriate Professionals

The Bhutan Medical and Health Council conducts licensing examinations and issues temporary registration certificates to successful expatriate candidates for one year, renewable. At the same time, the Council links with medical councils in other countries, especially those providing their health workers to Bhutan and maintains the list of health professional training institutions recognized by the respective countries.

The Maldives, with its high reliance on expatriate health professionals, manages inflow systematically. The Medical and Dental Council reviews whether the applicants’ qualifications and experience meet the Council’s criteria before granting preliminary registration as required for employment. Similar rules and procedures are applied to nurses.

In the Maldives, registration and licensure for domestically trained, native born-foreign trained, or foreign physicians or nurses who wish to practice in the Maldives are based on the same rules and procedures. The Maldives Medical and Dental Council is responsible for registration and licensing of doctors to practice. Foreign doctors need to take a pre-registration approval supported by evidence on qualification, work experience, and a certificate of good standing (CGS) issued by the medical licensing authority of the country where that doctor has been practicing in the last year. Medical graduates are required to produce evidence of English proficiency to the Council if English is not the medium of instruction. All need to sit an exam for professional practice to get a license granted by the Council. Practice without registration results in legal action. Maldivian nurses graduating from schools in-country or accredited schools elsewhere, who pass the national nursing license examination, can register and obtain their license to practice from the Nursing and Midwifery Council. Foreign nurses who come to work in the Maldives need pre-registration approval before sitting the license examination for professional practice. No re-licensing and continued professional developments are required by councils in Maldives.

In Indonesia, employment of foreign doctors and nurses is regulated by the Ministerial Decree 67 (2013) and for doctors also regulated by the Medical Council Decree 17 (2013). Expatriate health personnel are permitted to practice with the objective of transferring knowledge and skills to domestic health personnel on medical services, education and training, humanitarian and research in line with national health workforce needs. A resident’s permit from Ministry of Labour and a temporary license (one year, renewable) from the Medical Council or the National Nurses Association is required. The Indonesia Medical Practice Act and Nursing Act regulate how licenses are granted to both domestic and foreign medical and nursing personnel. Indonesian doctors who pass the national license exam receive certificates of competency and professional licenses from the Indonesian Medical Council. Nurses get these from the Indonesian Health Professional Council. Licenses are valid for 5 years; renewal is subject to gaining certain credits of continued medical or nursing education and successful competency exams. Similar competency tests are applied to foreign doctors and nurses for temporary licenses.

In Thailand, rules and procedures of health professional councils ensure equal rights and responsibilities between domestic and foreign health professionals. All applicants, national or native-foreign trained or expatriate professionals, have to sit and pass the national examination for license to practice issued by the Medical or Nursing and Midwifery councils and conducted by the Center for Medical Competency Assessment and Accreditation. The national examination can be an obstacle for expatriates who are not proficient in the Thai language, as the clinical part is conducted in Thai. There are no foreign professionals providing clinical services, though there are a few physicians and nurses on temporary license for humanitarian services, and not required to take national license examination.

Native born-foreign trained graduates need to hold a license from abroad that is recognized by the Medical Council. Foreign physicians need to hold valid license from countries of origin, and are issued temporary licenses, usually for less than a year, to work for a specific time-bound purpose, such as clinical research or humanitarian work without a license examination requirement. However, they must work under supervision of a fully registered physician.

Nurses that graduate from Thai schools or schools elsewhere that are recognized by the Council and who pass the national license examination are granted licenses to practice from the Nursing and Midwifery Council. Foreign nurses need to hold a valid license from the country of origin. Renewal of the license every 5 year is mandatory with a requirement of 50 credits of continued nursing education, such as attending training or conferences or completing on line distance courses approved by the Council. Similar to physicians, temporary licenses not requiring license examination are also issued by Thailand’s Midwifery council for nurses in humanitarian work.

In practice, work permits for professional practice, and employment visas are granted after the medical or nursing council approval in each country once candidates fulfill the required license examination.

### Policies in Destination Countries on Equal Employment for Expatriate Professionals

In Bhutan, foreign health workers are usually recruited for a period of 2-3 years, under service conditions as specified by the 2012 Bhutan Civil Service Regulation, which ensures they enjoy the same rights and benefits as domestic professionals. However, the contractual conditions in the Civil Service Regulation do not allow in- or post-service training for foreign professionals.

In the Maldives, there is equal treatment between domestic and foreign trained doctors and nurses. Incoming health personnel are hired, promoted and remunerated based on objective criteria such as qualification, years of experience, and scope of professional responsibility on the same basis as the domestically trained workers. However, high turnover results in the lack of continued professional development.

There are a limited number of international professionals in Indonesia, and they are employed with similar employment conditions to the national professionals.

Once professional licenses have been obtained, the Thai labour law provides equal employment conditions between expatriate and domestic professionals.

### Actions by Source Countries to Protect their Out-Migrating Professionals

Informal observation suggests very few Bhutanese health professionals leave to work abroad. There is no effective mechanism to trace those that do, and no obligation for them to report where they work once they are outside the Royal Civil Service.

An unknown number of health professionals leave the Maldives to practice elsewhere. Its small pool of health workers does not attract recruitment through either Government to Government (G to G) agreements or private recruiter arrangements.

In Indonesia, a surplus of training places and limited public sector positions for nurses and midwives triggered the Indonesian government to find employment for them abroad. This is conducted through systematic G to G and private to private (P to P) mechanisms. Under bilateral memorandum of understandings (MOUs) in the G to G agreements, the destination country specifies detailed requirements for numbers and skills, employment terms and conditions. However in practice numbers are determined mostly by an individual’s ability to obtain a license to practice in the destination countries.

In the Indonesia-Japan collaboration, rigorous requirements have been agreed between the 2 countries. Minimum qualifications for nurses are either 3-year diploma nurses with 2 years’ experience, or 4-year bachelor nurses with 1-year experience. In Indonesia, applicants have to pass a written test, psycho- and aptitude tests, video interview and a Japanese quiz. Nurses who qualify then need medical check-ups, 6-month Japanese language training and pre-departure orientation. In Japan, these Indonesia nurses take a 6-month advanced Japanese language course while temporarily working as a nurse assistant. Passing the Japanese national Kangoshi examination is required to become a registered nurse; failure to pass results in returning home. Indonesian registered nurses in Japan have similar pay and benefits as Japanese registered nurses.

The number of Indonesian nurses recruited via this mechanism increased from 166 in 2010 to 187 in 2014, fulfilling 50% of the Japanese demand for Indonesian nurses as negotiated between the 2 governments. Between 2008 and 2011, nearly 800 Indonesian nurses and care workers have entered Japan this way. However, difficulties in mastering Japanese results in a low success rate in the Kangoshi examination for registered nurses. By 2011, only 17 Indonesian nurses had passed it.^15,16^

Parallel to G to G, several private recruiters in Indonesia are actively recruiting nurses to work in around 45 countries. Applicants meeting requirements will be registered at the Ministry of Manpower to obtain an Identity Number for Indonesian workers and sign a placement agreement. In the destination country, they need to pass the local professional council’s license examination for professional practice.

Thailand does not have surplus of domestic health workers, and so has not developed policy and systematic management of out-migration. However, observations show an increasing trend of out-migration of young nurses who are English proficient, mostly through electronic recruitment by destination country employers or recruiters.

There are no specific mechanisms to support ‘circular’ migration: physician or nurse returnees, provided their professional licenses are valid, can work - but this is often in the private sector due to limited public sector vacancies and lower remuneration.

A common issue arising from this analysis is that source countries are unable to capture the number and profile of departing health professionals, except under the Indonesian G to G arrangements. This reiterates the importance of global solidarity especially on sharing migration information by destination countries with source countries as mandated by the Code.

Table 3 summarizes key findings on managing in- and out-migration of health professionals in these 4 countries.

**Table 3:** Summary Key Findings for Managing In-Migrate and Out-Migrate of Health Professionals

	**Bhutan**	**Indonesia**	**Maldives**	**Thailand**
HRH status: critical shortage	Yes	No	No	No
Local training capacities	Limited for physicians, adequate for nurses	Adequate	Limited for physicians, adequate for nurses	Adequate
Reliance on foreign trained doctors	Yes, but very small	No; government is starting to support nurses working outside Indonesia	Yes, substantial	No; some young nurses now out-migrate to other countries
Equal employment conditions	Yes for both domestic and foreign trained	Yes	Yes	Yes
Heath Professional licensing	Yes both domestic and foreign trained	Yes for both	Yes for both	Yes for both
Systematic arrangements for managing migration	Yes for in-migration, not for out-migration because numbers small	Yes, G to G arrangement with certain countries for out-migration	Yes for in-migration, through contracts; not for out-migration	Not for in-migration, nor for out-migration

Abbreviation: HRH, human resources for health.

## Discussion

Investment in the health workforce is critical to improve access to health services and achieve UHC.^17^ Within the Sustainable Development Goal for health, UHC underpins achievement of other targets such as improved child and maternal mortality and universal access to sexual and reproductive health services. Having sufficient numbers of skilled and motivated health workers requires significant investment in two inter-related areas: health professional education reform, to ensure the workforce is ‘fit for purpose’^18^ and in actions that retain health workers in places where they are needed most^19^ - both in-country, and within the country in rural hard to reach areas. Addressing push factors and having effective implementation of the Code will support retention in country.

We find that 4 countries with different health workforce profiles and needs are responding differently in the ways they manage in- and out-migration, but certain regulatory practices are common to all. For in-migration, a license to practice issued by the national professional council is mandatory, and the profiles of incoming professionals are well captured by these councils. Licenses are more commonly temporary rather than permanent. Language proficiency, commonly required by destination countries, is the major barrier for foreign trained health professionals in passing license examinations. The review also notes that in cases where governments publicly subsidize medical students to be trained overseas, policies to compel the return of medical graduates are needed. For example, in the Maldives a more effective enforcement of the training contract, together with sanctions if it is broken, would be useful.

Some good practices are noted. All countries apply equal legal and employment practices between expatriate and domestically trained professionals. Indonesia has the most systematic out-migration management of nurses - through G to G agreements, regulated private to private mechanisms, and pre-departure orientation.

This study provides new understanding of the situation in countries in SEA, it indicates that systematic arrangements between source and destination country governments is useful in moderating migration and protecting the out-migrating professionals.

A few policy implications for other countries emerge. In countries with limited local training capacities, governments need to ensure that publicly subsidized medical students trained abroad return home to serve in their country health systems. This can be achieved through positive incentives and also legal sanctions for non-adherence. This will gradually reduce the reliance on the use of foreign professionals.

Formal agreements between source and destination governments (the G to G agreements) are a good practice which can have several benefits. They can help to ensure that out-migrating professionals have similar employment rights and benefits to domestically trained professionals. G to G agreements can also moderate the number and qualifications of out-migrating professionals, and help reduce the negative consequences on source country health systems. Private recruiters should be registered, aware of the Code and prevented from using unethical recruitment practices.

## Conclusion

This study does not provide particularly different findings from earlier studies on managing health worker migration, but it does expand the body of knowledge on what is happening in countries in Asia by analyzing current policy and practice in 4 countries that have been relatively under examined in this context. It also reaffirms the critical importance of the Global Code of Practice.

The main policy instruments being used to ethically manage in-migration are mandatory initial professional licensing and equal employment procedures, and these are found in all 4 countries. Re-licensing requirements are less common. The size of out-migration is unknown, except in Indonesia, where there is some information through Government to Government agreements. Destination countries need to share information on health worker migration source countries, as mandated by the Code. Source country policies to protect their out-migrating professionals are also hampered by the fact that most out-migrating professionals are outside government to government agreements.

The analysis reaffirms that systematic arrangements between source and destination country governments are useful in protecting health system integrity, moderating migration, and protecting out-migrating professionals. The Global Code of Practice provides a valuable framework to promote this, and remains an essential platform for more effective collaboration between source and destination countries.

## Ethical issues

No ethical approval was required as this manuscript employed secondary data for the analysis.

## Competing interests

The authors declare that they have no competing interests.

## Authors’ contributions

VT, PT, AST, KS, YC, SH, and NP developed the common protocol together. All relevant documents in each country were reviewed and synthesized by the country authors. VT, PT, and NP performed the cross-country data analysis. VT, PT, AST, KS, YC, SH, and NP jointly designed and drafted this manuscript. All authors read and approved the final manuscript.

## Authors’ affiliations

^1^International Health Policy Program, Ministry of Public Health, Nonthaburi, Thailand. ^2^WHO South East Asia Region, Delhi, India. ^3^Ministry of Health, Jakarta, Indonesia. ^4^Public Health Technical Office, Ministry of Public Health, Nonthaburi, Thailand. ^5^Ministry of Health, Thimphu, Bhutan. ^6^Ministry of Health, Malé, Maldives. ^7^Healthcare Accreditation Institute (Public Organization), Nonthaburi, Thailand.

## Key messages

**Implications for policy makers**

- A mandatory license to practice issued by the national professional councils should be maintained to ensure quality of service, patient safety, and personnel safety for both domestic and in-migrating professionals.
- Policies requiring government sponsored medical students trained overseas to return home are needed. This could be done through more effective enforcement of the training contract, together with sanctions if it is broken.
- More systematic out-migration management of health professionals, through government to government agreements, could create a win-win situation as well as provide greater benefits and opportunities for health workers.
- To effectively capture the number and profile of outflow health professionals, there is a need for improving health workforce data capture and better sharing of migration information between source and destination countries.
- The World Health Organization (WHO) Global Code is relevant and useful for addressing health workforce development. All countries should support its implementation as well as regularly reporting on progress.

**Implications for public**

All 4 countries (Bhutan, Indonesia, Maldives, and Thailand) have committed to progressing towards universal health coverage (UHC). A sufficient, well-performing health workforce is essential for the achievement of UHC. Several policy instruments are being used, in line with recommendations of the World Health Organization (WHO) Global Code: policies by destination countries to ensure expatriate professional quality through licensing and equal practice, and source and destination country collaborative actions to ensure the out-migrating professionals are equally treated by destination country systems. A common problem is that source countries cannot capture the number and profile of out-migrating professionals, except through Government to Government arrangements as in Indonesia. This reiterates the importance of global solidarity especially on the need for destination countries to share health worker migration information with source countries, as mandated by the Code. This study reinforced that the Code is still relevant and is an essential platform for effective collaboration between destination and source countries to strengthen health workforce development.
